# Impacts of Endoscopic Gastroesophageal Flap Valve Grading on Pediatric Gastroesophageal Reflux Disease

**DOI:** 10.1371/journal.pone.0107954

**Published:** 2014-09-18

**Authors:** Kai-Chi Chang, Jia-Feng Wu, Wei-Chung Hsu, Bor-Ru Lin, Huey-Ling Chen, Yen-Hsuan Ni

**Affiliations:** 1 Department of Pediatrics, National Taiwan University Hospital and National Taiwan University College of Medicine, Taipei, Taiwan; 2 Department of Otolaryngology, National Taiwan University Hospital and National Taiwan University College of Medicine, Taipei, Taiwan; 3 Departments of Integrated Diagnostics and Therapeutics, and Internal Medicine, National Taiwan University Hospital and National Taiwan University College of Medicine, Taipei, Taiwan; Cincinnati Children's Hospital Medical Center, University of Cincinnati College of Medicine, United States of America

## Abstract

**Background:**

Gastroesophageal flap valve (GEFV) endoscopic grading is reported to be associated with gastroesophageal reflux disease (GERD) in adults; however its role in pediatric groups remains unknown. This study aimed to investigate the significance of GEFV grading and the associations to multichannel intraluminal impedance and pH monitoring (MII-pH) in children with GERD.

**Methods:**

A total of 48 children with GERD symptoms who received esophagogastroduodenoscopy and MII-pH monitoring were enrolled. The degree of GEFV was graded from I to IV according to the Hill classification, and classified into two groups: normal GEFV (Hill grades I and II), and abnormal GEFV (Hill grades III and VI). Endoscopic findings and MII-pH monitoring were analyzed among the groups.

**Results:**

Thirty-six patients had normal GEFV while 12 had abnormal GEFV. The presence of erosive esophagitis was significantly more common in the patients with abnormal GEFV (*p* = 0.037, OR 9.84, 95% CI 1.15–84.42). Pathological acidic gastroesophageal reflux (GER) determined by MII-pH was more prevalent in the patients with loosened GEFV geometry (*p* = 0.01, OR 7.0, 95% CI 1.67–27.38). There were significant positive correlations between GEFV Hill grading I to IV and the severity of erosive esophagitis (*r* = 0.49, *p*<0.001), percentage of supine acid reflux (*r* = 0.37, *p* = 0.009), percentage of total acid reflux (*r* = 0.3284, *p* = 0.023), and DeMeester score (*r* = 0.36, *p* = 0.01) detected by pH monitoring. In the impedance study, GEFV Hill grading also positively correlated to median number of acid reflux events (*r* = 0.3015, *p* = 0.037).

**Conclusions:**

GEFV dysfunction highly associated with acid GER and severe erosive esophagitis. An abnormal GEFV is a sign of acid GER in children.

## Introduction

The gastroesophageal junction acts as a barrier against the retrograde flow from the stomach. Several anti-reflux mechanisms contribute towards its proper function, including the coordination of diaphragm crural fibers, lower esophageal sphincter (LES), and the intra-abdominal portion of the esophagus, all of which combine to maintain an anti-reflux effect. Moreover, the flap valve formed by gastric cardiac sling musculature also plays an important role as a gate against anti-gastric retrograde flow [1]–[4]. This collar musculature is located at the gastric cardiac portion maintaining the acute angle of His [5]. During gastric inflation, the gastroesophageal flap valve (GEFV) provides a pressure gradient against the reflux of stomach contents [6]. The flap valve was first described in cadavers by Thor et al. in 1987 [7], and later a grading system to evaluate reflux was proposed by Hill et al [5], [8].

Gastroesophageal reflux disease (GERD) is presented when the reflux of gastric contents causes troublesome symptoms and/or complications. The incidence rate of GERD per1000 person- years is approximately 0.84 in pediatric patients aged 1–17 years in the UK with an increasing trend [9]. The symptoms of gastroesophageal reflux in children are more nonspecific than in adults. Affected children may present with vomiting, dysphagia, easy choking, and other extra-esophageal presentations such as cough, apnea, recurrent pneumonia, and even failure to thrive [10]–[12]. Since children suffer from such a bothering health-damaging issue, more and more diagnostic tool assessing gastroesophageal reflux (GER) are launched in these years. Combined multichannel intraluminal impedance and pH monitoring (MII-pH) is one of the new technique allowing for the detection of GER episodes irrespective of the pH value and reflux composition. It is superior to conventional pH monitoring for the evaluation of GER, and especially for the nonacid type [13]–[15].

In adults, the degree of GEFV dysfunction has been reported to be positively related to acid reflux [16]. To the best of our knowledge, no previous study has reported the assessment of this endoscopic finding in children and the clinical implications. The aim of this study, therefore, was to evaluate GEFV Hill grading in symptomatic children, and to assess its correlation with GERD as evidenced by MII-pH monitoring.

## Methods

### Patients

This study was conducted with a retrospective manner in children with GERD at the Department of Pediatrics, National Taiwan University Hospital from January 1, 2010 through January 31, 2014. The patients were referred for evaluations due to either respiratory symptoms/signs (cough, asthma, hoarseness, stridor) or gastrointestinal diseases (nausea, vomiting, regurgitation, dysphagia, heartburn sensation). Children who underwent esophagogastroduodenoscopy (EGD) within 3 days before MII-pH monitoring examination, and never received any acid suppression therapy were eligible for inclusion in the study. GEFVs were graded from I to IV by retrograde endoscopic findings [6], [8]. Multiple impedance parameters were analyzed to define reflux episodes.

### Ethics Statement

The written informed consents were obtained from the parents or guardians of the children. The study protocol was approved by the Ethics Committee of National Taiwan University Hospital’s Institutional Review Board.

### Multichannel intraluminal impedance pH monitoring

Each MII-pH study was performed using an ambulatory polyurethane catheter incorporating six impedance amplifying electrodes 2.0 cm apart and a single pH sensor, positioned 5.0 cm above the tip (Medical Measurement Systems, Inc., Netherlands). The catheter was introduced transnasally and placed with the pH sensor 3–5 cm above the LES confirmed by chest radiography. The MII-pH electrodes were connected to an ambulatory recorder. All patients were allowed to maintain their usual activities, regular diet, and sleep routines. This study was carried out for at least 20 hours for each subject. During the study period, the patient or the caregiver was instructed to press event buttons and to keep a diary to record meal times, posture changes, and symptoms. At the end of the measurements, recordings within the device were uploaded to a computer and analyzed manually by the physician using a dedicated software program (Ohmega, Virtual Instructor Program, Medical Measurement Systems, Inc.).

While impedance can detect voltage and electrical current changes in consecutive sensors positioned along the catheter, the impedance wave was designed to change inversely to ionic concentration. Air with a relatively low ionic content results in a high impedance wave, and fluid with a higher ionic content results in a low wave. This principle provides an essential advantage in the assessment of the flow direction of bolus passage.

The impedance recording was analyzed according to the protocol recently proposed by the European Society for Pediatric Gastroenterology, Hepatology, and Nutrition (ESPGHAN) [17]. A reflux episode was defined as sequential, progressive drops of impedance to at least 50% of the initial value in the most distal channels, and proceeding retrograde across two or more proximal channels. Reflux episodes were considered complete when the voltage value returned to baseline [18], [19].

With regards to pH recording data, catheter acidification by the surrounding contents causing the pH value to drop lower than 4 was classified as an acidic episode, and pH values remaining above 4 were classified as non-acidic episodes. A widely used pH reflux composite score was calculated based on the DeMeester criteria including both percent times in upright and supine acid reflux, total percent of time in acid reflux, duration of the longest acid reflux episode, the number of acid reflux episodes >5 minutes, and total number of reflux events. Pathological acid gastroesophageal reflux in our study population was defined based on the finding of total percent of time in acid reflux >4.2% or DeMeester score >14.7 [20], [21]. One of the authors (J-FW) interpreted all data of the MII-pH monitoring and reported the results.

### Endoscopy

**Figure 1:**
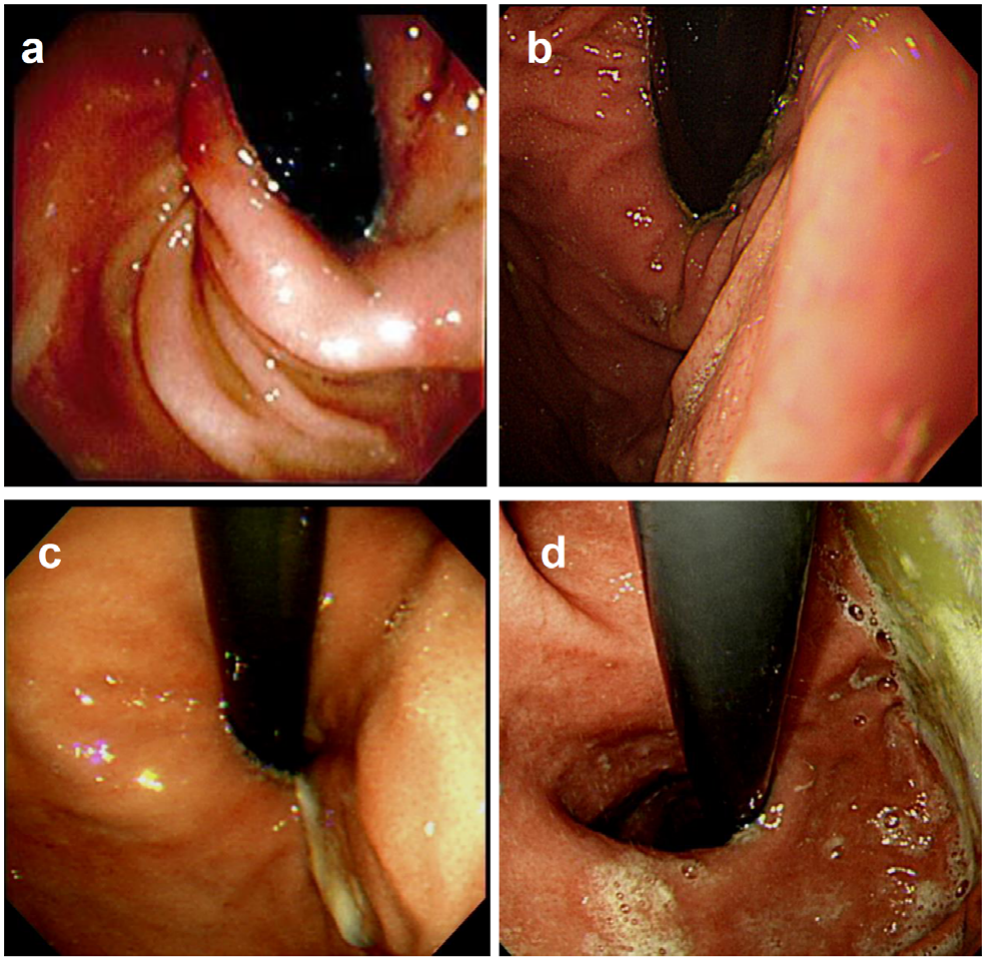
Retroflex view of the gastroesophageal flap valve. (a) Grade I. The prominent fold of tissue along the lesser curvature apposed closely to the endoscope. (b) Grade II. The fold was present but less well defined than in grade I, and some periods of opening and rapid closing around the endoscope were found. (c) Grade III. The fold was not prominent and often failed to close around the endoscope, gripping it tightly. (d) Grade IV. There was no fold and the lumen of the esophagus was open. The squamous epithelium of the esophagus could be seen below.

Flexible EGD was performed in each patient under general or local anesthesia, and the esophagus, stomach, and duodenum were examined with optimal visualization. The severity of erosive esophagitis was graded according to Los Angeles (LA) classification. In addition, we carefully examined the gastroesophageal junction to assess the geometry of the GEFV, using a retroflexed view during gastric inflation. The GEFV was graded from I to IV according to the Hill classification (Figure 1) [6], [8]. We defined GEFV grades I and II as the normal GEFV, and grades III and IV as the abnormal GEFV [22]–[24]. One author (K-CC) retrospectively reviewed all the EGD pictures of the enrolled subjects and re-graded the erosive esophagitis and GEFV, according to the LA and Hill classifications separately. The results of MII-pH monitoring and endoscopic findings were independently interpreted.

### Statistical analysis

We used the *X* ^2^ test and Fisher’s exact test to examine differences in categorical variables between the two patient groups. The Mann-Whitney U test was applied to test the differences in median/25^th^−75^th^ percentage values in the continuous variables. Linear regression analysis was performed to estimate the correlations between the variables of MII-pH monitoring and Hill classification. All *p* values were 2-sided, and a *p* value less than 0.05 were considered to be significant. Statistical calculations were performed with SPSS version 19.0 for Windows software (SPSS, Chicago, IL, USA).

## Results

### Patient

A total of 48 patients (Male: Female = 2424) were enrolled in this study. The median age of these children was 6.2 years. The most common indications for receiving these examinations were vomiting and dysphagia. Demographic data and clinical characteristics were summarized in Table 1. Endoscopic erosive esophagitis was observed in 30 patients; 11 of whom with grade A, 5 with grade B, 11 with grade C, and 3 with grade D. Sixteen patients were detected with total percentage of time in acid reflux (%) >4.2% or DeMeester score >14.7 by MII-pH monitoring. Among all of the 48 subjects, 36 patients were categorized as normal GEFV group (5 of GEFV Hill grade I, 31 of grade II), and 12 patients as abnormal GEFV group (8 of grade III, 4 of grade IV).

**Table 1:** Patient profiles and endoscopic findings according to the gastroesophageal flap valve grades.

	Gastroesophageal flap valve		
	Hill grade I & II(n = 36)	Hill grade III & IV(n = 12)	P value
**Demographic data**			
Age, median (25–75%) years	5.3 (1.5–12.6)	12.4 (3.9–13.6)	0.25
Body weight, median (25–75%)	17 (8.75–36.5)	34.9 (14.8–51.1)	0.19
Body height (cm), median (25–75%)	117.5 (84–147)	146 (100–162)	0.17
Male, n(%)	18 (50%)	6 (50%)	1
Hiatal hernia, n(%)	3 (8.3%)	3 (25.0%)	0.13
Laryngomalacia, n(%)	3 (8.3%)	1 (8.3%)	1
Neurological disease, n(%)	17 (47.2%)	4 (33.3%)	0.31
Cardiologic disease, n(%)	5 (13.8%)	1 (8.3%)	0.53
**Endoscopic erosive esophagitis** †			
No esophagitis	17	1	
A	10	1	
B	2	3	0.004
C	6	5	
D	1	2	
**pH moniter** ‡			
Upright acid reflux (%)	2.0 (0.3–4.9)	4.5 (1.7–22.4)	0.10
Supine acid reflux (%)	0.3 (0–2.5)	7.0 (2.6–23.8)	<0.001
Total acid reflux (%)	1.8 (0.4–4.0)	10.0 (1.7–17.3)	0.02
Longest acid reflux (min)	4.6 (1.3–19.0)	30.5 (3.1–59.1)	0.07
Events of acid reflux >5 mins	0.5 (0–2)	2.5 (0–11)	0.07
DeMeester score	7.2 (1.9–13.6)	36.9 (6.9–66.2)	0.01
**Impedance** ‡			
Acid reflux (events)	9.5 (2–20.5)	21.5 (7.0–39.5)	0.05
Nonacid reflux (events)	1.0 (0–2)	1.5 (0–6.5)	0.39
Liquid reflux (events)	18.0 (8.5–28.5)	16.0 (5.5–24.5)	0.51
Mixed liquid-gas reflux (events)	14.5 (4.5–48)	28.5 (10–59)	0.33
Total reflux (events)	42.0 (18–76)	53.0 (19–85.5)	0.47

Fisher exact test or chi-square test was used for categorical data. Continuous non-parametric variables were compared by Mann-Whitney u test.

† Los Angeles classification grade.

‡ The values of pH monitor and Impedance findings were expressed as median (25%–75% range).

### Correlation between GEFV grade and MII-pH monitoring, endoscopy

For the pH monitoring, the percentage of supine acid reflux and total acid reflux were significantly increased in the abnormal GEFV group (*p*<0.001, and *p* = 0.02, respectively). The median DeMeester score was also significantly higher in the abnormal GEFV group (36.9, 6.9–66.2) than that in normal GEFV group (7.2, 1.9–13.6; *p* = 0.01). The ratio of subjects fulfilling the criteria of pathological acid gastroesophageal reflux with total acid reflux (%) >4.2% or DeMeester score >14.72 was greater in abnormal GEFV group than normal group (66.7% and 22.2%, respectively, *p* = 0.01, OR 7.0, 95% CI 1.67–27.38) (table 2). In addition, GEFV grading was positively correlated with supine acid reflux (*r* = 0.37, *p* = 0.009), total acid reflux (*r* = 0.3284, *p* = 0.023), and the DeMeester score (*r* = 0.36, *p* = 0.01).

**Table 2:** Logistic regression analysis of the association between GEFV and erosive esophagitis, pathological gatroesophageal reflux.

	Gastroesophageal flap valve			
	Hill grade I & II(n = 36)	Hill grade III & IV(n = 12)	OR (95% CI)	P value
Endoscopic erosive esophagitis	19 (52.8%)	11 (91.7%)	9.84 (1.15–84.42)	0.037
Total acid reflux (%) >4.2% or DeMeesterScore >14.7	8 (22.2%)	8 (66.7%)	7.0 (1.67–29.38)	0.008

Upright acid reflux, duration of the longest acid reflux episode, and the number of acid reflux events >5 minutes also showed higher trends in the abnormal GEFV group than in normal GEFV group(upright acid reflux, *p* = 0.1; duration of longest acid reflux, *p* = 0.07; the number of acid reflux events >5minutes, *p* = 0.07).

With regards to impedance, the abnormal GEFV group had a significantly higher median number of acid reflux events (abnormal GEFV group *vs.* normal GEFV group, 21.5 *vs.* 9.5, *p* = 0.05). In addition, GEFV gradings also positively correlated to the median number of acid reflux events (*r* = 0.3015, *p* = 0.037). However, there were no significant differences between the normal and abnormal GEFV groups in terms of other parameters including nonacid reflux, liquid reflux, mixed liquid-gas reflux, and total impedance events per hour detected.

Among the 48 patients, the presence of erosive esophagitis was significantly more prominent in the patients with abnormal GEFV than in those with normal GEFV (*p* = 0.037, OR 9.84, 95% CI 1.15–84.42) (table 2). The GEFV Hill classification I to IV was markedly positively correlated with the presence and severity of erosive esophagitis (*r* = 0.49, *p*<0.001).

### Correlation of treatment responses with GEFV grading

Proton pump inhibitors (PPIs) are the mainstay of treatment of GERD [13]. For those children with erosive esophagitis found by EGD or acid gastroesophageal reflux detected by MII-pH monitor, PPI was used to suppress gastric acid secretion. Among the 48 patients, 22 of 36 cases in the normal GEFV group and all of 12 in the abnormal GEFV group received either lansoprazole or esomeprazole, and were monitored at outpatient clinics (p = 0.01, OR 1.64, 95% CI 1.32–2.25). These two PPIs were given with a standard dose of 0.7–3.0 mg/kg daily. An effective treatment response was defined as subjective symptom resolution (n = 31, 91.2%) or endoscopic esophageal mucosal healing within 2 months (n = 3, 8.8%).

The treatment response to standard doses of the PPIs in the normal GEFV group was significantly better than that in the abnormal GEFV group (normal group *vs.* abnormal group: n = 22/22, 100% *vs.* n = 7/12, 58.3%, *p* = 0.003, OR 0.58, 95% CI 0.36–0.94). Doubling the PPI dose to a twice-daily manner for the 5 subjects with abnormal GEFV who initially failed to respond to a standard dose of PPIs revealed subsequent therapeutic advantages. None of the treatment subjects in normal GEFV group needed to receive the double dose of PPI. Five of 14 patients in the normal GEFV group who did not use PPIs received prokinetic regimens or mucosal protective agents, and demonstrated symptom relief.

## Discussion

In the present study, the prevalence and severity of erosive esophagitis indicating macroscopic mucosal damage increased significantly with the progressive loss of sling structure of the flap valve. Prior studies in adults have also demonstrated similar findings [16], [25], [26]. The present study is the first report to confirm the relationship between altered GEFV grading and prevalence of erosive esophagitis in children.

Gastroesophageal reflux in the acidic phase was also found to be related to an abnormal GEFV. This finding is consistent with previous studies in adults where the percentage of time with a pH below 4.0 increased in a stepwise fashion with the altered geometry of the GEFV [23]–[25]. However, in prior studies, esophageal reflux defined by ambulatory 24-hour pH monitoring only detected acid reflux. Without the aid of MII, non-acid events were ignored and distinct components such as gas or liquids could not be well differentiated. Using pH monitoring in combination with MII, we assessed reflux with all pH values and variable ionic contents accurately, which demonstrated that the degree of GEFV had no obvious correlation with regards to non-acid reflux and different intra-esophageal contents. In our presented cases, the DeMeester score and total percentage of time in acid reflux (%) in abnormal GEFV group were markedly higher than 14.7 and 4.2%, which might be attributed to acid GER [27].

In addition, we demonstrated that the prevalence of acid reflux in a supine rather than in an upright position correlated more significantly with loosening of the GEFV. It was speculated that the gastric contents in the supine position with a lower gravity effect could reflux more easily in the abnormal GEFV group due to attenuation of the collar-sling musculature. Previous studies suggested that patients with bipositional reflux in which supine reflux was predominant has a high potential of a completely defective LES with a larger gastroesophageal junction diameter [28], [29]. Gastric fluid would consequently be able to flow into the esophagus in the supine position but could not be cleared effectively within a short period, resulting in longer esophageal acid exposure and mucosal damage [28], [29].

The results of our survey also showed that gastroesophageal junction morphology may aid in the estimation of the effectiveness of anti-reflux treatment in GERD patients. Our subjects with GEFV Hill grade III and IV did not respond well to a once daily standard dose of lansoprazole or esomeprazole. Doubling the PPI dose in a split fashion (morning and night) subsequently alleviated their symptoms. In contrast, none of the subjects with GEFV Hill grade I or II was indicated for a doubling of the PPI dose. This is consistent with previous reports which suggested that an abnormal GEFV configuration could serve as an independent factor predicting a poor response to PPI therapy [30], [31]. Taken together, we suggest that GEFV grading is a prognostic factor for the long-term medical management of GERD.

In conclusion, endoscopic grading of the GEFV had a positive correlation with erosive esophagitis and some parameters of MII- pH monitoring in children. Our findings suggest that evaluation of the GEFV can provide useful information to help predict the presence of acid gastroesophageal reflux and its associated adverse events in children. In addition, loosening GEFVs may correlate with poor responses to PPI regimens for children with GERD.
